# Reducing salt in food; setting product-specific criteria aiming at a salt intake of 5 g per day

**DOI:** 10.1038/ejcn.2015.5

**Published:** 2015-02-18

**Authors:** M Dötsch-Klerk, W PMM Goossens, G W Meijer, K H van het Hof

**Affiliations:** 1 Unilever Research and Development Vlaardingen, Vlaardingen, Netherlands

## Abstract

**Background/Objectives:**

There is an increasing public health concern regarding high salt intake, which is generally between 9 and 12 g per day, and much higher than the 5 g recommended by World Health Organization. Several relevant sectors of the food industry are engaged in salt reduction, but it is a challenge to reduce salt in products without compromising on taste, shelf-life or expense for consumers. The objective was to develop globally applicable salt reduction criteria as guidance for product reformulation.

**Subjects/Methods:**

Two sets of product group-specific sodium criteria were developed to reduce salt levels in foods to help consumers reduce their intake towards an interim intake goal of 6 g/day, and—on the longer term—5 g/day. Data modelling using survey data from the United States, United Kingdom and Netherlands was performed to assess the potential impact on population salt intake of cross-industry food product reformulation towards these criteria.

**Results:**

Modelling with 6 and 5 g/day criteria resulted in estimated reductions in population salt intake of 25 and 30% for the three countries, respectively, the latter representing an absolute decrease in the median salt intake of 1.8–2.2 g/day.

**Conclusions:**

The sodium criteria described in this paper can serve as guidance for salt reduction in foods. However, to enable achieving an intake of 5 g/day, salt reduction should not be limited to product reformulation. A multi-stakeholder approach is needed to make consumers aware of the need to reduce their salt intake. Nevertheless, dietary impact modelling shows that product reformulation by food industry has the potential to contribute substantially to salt-intake reduction.

## Introduction

There is increasing public health concern regarding high sodium intake. The World Health Organization (WHO) recently reviewed the guideline for sodium intake,^1^ and confirmed their earlier conclusions regarding the adverse effects of high sodium intakes particularly on blood pressure, and consequently on the risk of cardiovascular disease.^2^

Sodium intake largely originates from sodium chloride in the diet, commonly referred to as salt. In many countries, the average current daily salt intake is between 9 and 12 g,^3, 4^ whereas WHO recommends <5 g of salt (2000 mg of sodium) per day.^1^ In the industrialised countries, at least 75% of the salt intake comes from processed food products.^3, 4^ In addition, in many non-industrialised countries, salt intake exceeds recommendations; in these countries, salt added during preparation or at the table at home are the main contributors to daily salt intake, contributing up to 75%.^5^

International health authorities advocate salt reduction in foods as a cost-effective strategy to improve the public health. Currently, several relevant sectors of the food industry are engaged in salt reduction, despite the fact that reformulation of food products may imply additional costs for product development. However, different stakeholders need to work together in order to reduce intakes successfully, and consumers need to be encouraged to buy salt-reduced products and reduce their use of discretionary salt.

As a global food company, Unilever is continuously working to improve the nutritional quality of the product portfolio. Despite challenges with regard to consumer acceptability of salt-reduced food and technological challenges (for example, salt has a role in preservation (dressings), and structuring of products (bread, meat)), the aim is to reduce, systematically, the amount of salt across the portfolio, in an effort to help consumers meet globally recommended daily salt intakes. In 2003, the Nutrition Enhancement Programme was launched to evaluate and improve the nutritional quality of the Unilever foods portfolio.^6^ In line with WHO's Global Strategy on Diet, Physical Activity and Health, sodium was selected as one of the key nutrients to focus on next to saturated fat, trans fat and (added) sugar.^7^

The salt reduction strategy is now further intensified as part of the Unilever Sustainable Living Plan.^8^ Within the plan, two sets of globally applicable product group-specific sodium criteria were developed to guide further product reformulation. The first set aimed at reducing salt levels in foods to help consumers reduce their intake towards an interim intake goal of 6 g/day. The second set aims at further reducing intakes on the longer term towards 5 g/day, as recommended by WHO. A two-step approach was applied to assess the potential impact of cross-industry food product reformulations on salt intake in populations. In the first step, the impact of test criteria was estimated using typical daily diets in various countries. In a second step, the potential impact of the final product criteria was evaluated via scientific modelling using population intake data of the United States (US), United Kingdom (UK) and the Netherlands (NL). In this paper, the development and impact evaluation of these criteria are described.

## Materials and Methods

### Development of the salt targets

Various sets of criteria for salt or sodium reduction have been developed by health authorities or governments, but these criteria do have their limitations. Criteria are often set to differentiate between products currently on the market in order to determine eligibility for claims,^9^ marketing to children^9^ or health logos,^10^ which is different from steering long-term product reformulation. Some criteria are pragmatically set and embedded in the local nutrient profiling systems, which are not globally applicable.^11^ Some sets of criteria are not covering the entire diet, making it difficult to get overall salt-intake levels towards a certain intake goal.^12^ Some systems do include many different criteria for sub-categories of food, making application very complex.^12^ Moreover, most of these criteria have been set pragmatically without a clear science-based rationale or impact evaluation through dietary intake modelling. The lack of a gold standard and/or globally applicable product targets stimulated Unilever to develop its own set of product group-specific sodium criteria aiming to reduce salt levels in foods over time to ultimately help consumers reducing their intake towards population intake targets.

### Defining the intake goals

International, generally accepted, dietary recommendations formed the basis for the development of the salt reduction criteria. Criteria were set to target a dietary intake of 6 g of salt (2400 mg of sodium), and a further reduction towards 5 g of salt (2000 mg of sodium). The first target is in line with current recommendation used in many countries;^13^ the second target is the recommendation of the WHO.^1^

### Conversion of intake goals into product group-specific targets

When developing the criteria, a total diet approach was taken. Thus, product group-specific sodium criteria were set for all product groups contributing to sodium intake and not only product groups relevant for Unilever. Absolute sodium criteria were defined, rather than relative reduction targets. Product group-specific absolute criteria allow taking into account the composition and the contribution of product groups to overall daily salt intake.

Different sets of sodium test criteria were developed, taking the criteria used in Unilever's Nutrition Enhancement Programme,^6^ the sodium criteria applied in various countries (for example, UK)^11^ or international nutrient profiling systems (for example, International Choices Programme)^10^ as a starting point. The potential impact of these test criteria on total salt intake was evaluated through daily menu modelling, as described below.

### Impact evaluation of test criteria using typical daily menus (step 1)

Product group-specific sodium criteria were tested using typical daily menus.^14^ For several countries, average salt intakes from national surveys were converted into three typical daily menus per country. To provide a global representation, daily menus were created for the Netherlands,^15^ Spain,^16^ Greece,^17^ US,^18^ South Africa^19^ and China.^20^ Details about these daily menus are described in Roodenburg *et al.*^14^ In these menus, the contribution of food products to daily salt intake in the context of their role within a typical menu is taken into account—focusing on how often and how much salt-containing products consumers typically eat. Salt-intake levels used for the modelling did not include intake from discretionary salt, except for Spain where salt intake could be based solely on 24- h urine excretion, and the US, where salt used during cooking was included in the recipes of prepared foods. With these menus, the impact of product group-specific sodium criteria on total daily salt intake was crudely estimated by adjusting the sodium levels of all non-complying products in these menus to the levels of the test criteria. Finally, the average total daily salt intake after adjusting the sodium product values was compared with salt recommendations to evaluate the effectiveness of the sodium test criteria. This iterative process finally led to the 6 and 5 g/day product group criteria, as shown in Table 1. More detailed information on the criteria is available as Supplementary information.

### Impact evaluation of final criteria on population salt intake (step 2)

To have a more robust insight in the potential impact of the product group-specific sodium criteria on population salt intake, additional modelling was conducted by using the complete food consumption survey data from the US,^21^ UK^22^ and NL.^23^ The sodium content in food products, listed in the composition tables^24, 25, 26, 27^ attached to the surveys, was modified to simulate sodium-specific product reformulation. An overview of the food consumption surveys^21, 22, 23^ and food composition tables^24, 25, 26, 27^ used in the assessments is presented in Table 2. Discretionary salt intake was not included in the surveys, except for the US survey where salt used during cooking was included in the recipes of prepared foods. Analyses were performed with Crème Food between December 2011 and March 2012.

For each of the countries, three scenarios were analysed:

- Population salt intake based on the original survey data (as measured).
- Hypothetical population salt intake following the reformulation to meet the product group-specific sodium criteria aiming at 6 g salt/day.
- Hypothetical population salt intake following the reformulation to meet the product group-specific sodium criteria aiming at 5 g salt/day.

To calculate the ‘as measured' overall population salt intake in the surveys, a standard assessment was performed in which no nutrient levels were altered in the original food composition data. To model the impact of the 6 and 5 g salt/day criteria, sodium levels in the food composition data were modified to simulate reformulation, assuming that all food products included in the survey would be compliant with the relevant criteria. Sodium values in the food composition tables for the products that were not compliant to the criteria (Table 1) were modified to meet these criteria. For example, sodium levels of all soups consumed in the survey that were not complying with the 6 g/day criterion of 360 mg/100 g and/or 5 g/day criterion of 265 mg/100 g were adjusted to these levels. Products with sodium levels that were already compliant to the sodium criterion for the relevant sodium product group were not modified.

For all scenarios, median daily salt intake was calculated for the total population and for a subgroup of high-salt users. This population subgroup was identified because of their relatively higher risk of high blood pressure and subsequent cardivascular disease risk, and therefore they are expected to benefit more from salt-intake reduction. The high-salt user subgroup was defined as the quartile of the population with the highest salt consumption. In addition to median intakes, the percentage of the population compliant with overall intake targets was calculated.

To analyse the different scenarios, it was necessary to align food groups in the food composition tables with the food groups for which the different product-specific criteria were defined (see Table 1). All foods that could not be categorised into the specific product groups (USA, 30% UK, 22% and NL, 41% of products listed in the country-specific composition table) were modelled to comply with the ‘generic' sodium criteria (for example, beverages, milk products, bread toppings, fruits and vegetables, potatoes and grains). For product groups with two criteria, either the criterion in x mg/100 g or x mg/kcal had to be met.

## Results

### Impact evaluation of test criteria using typical daily menus (step 1)

**Figure 1:**
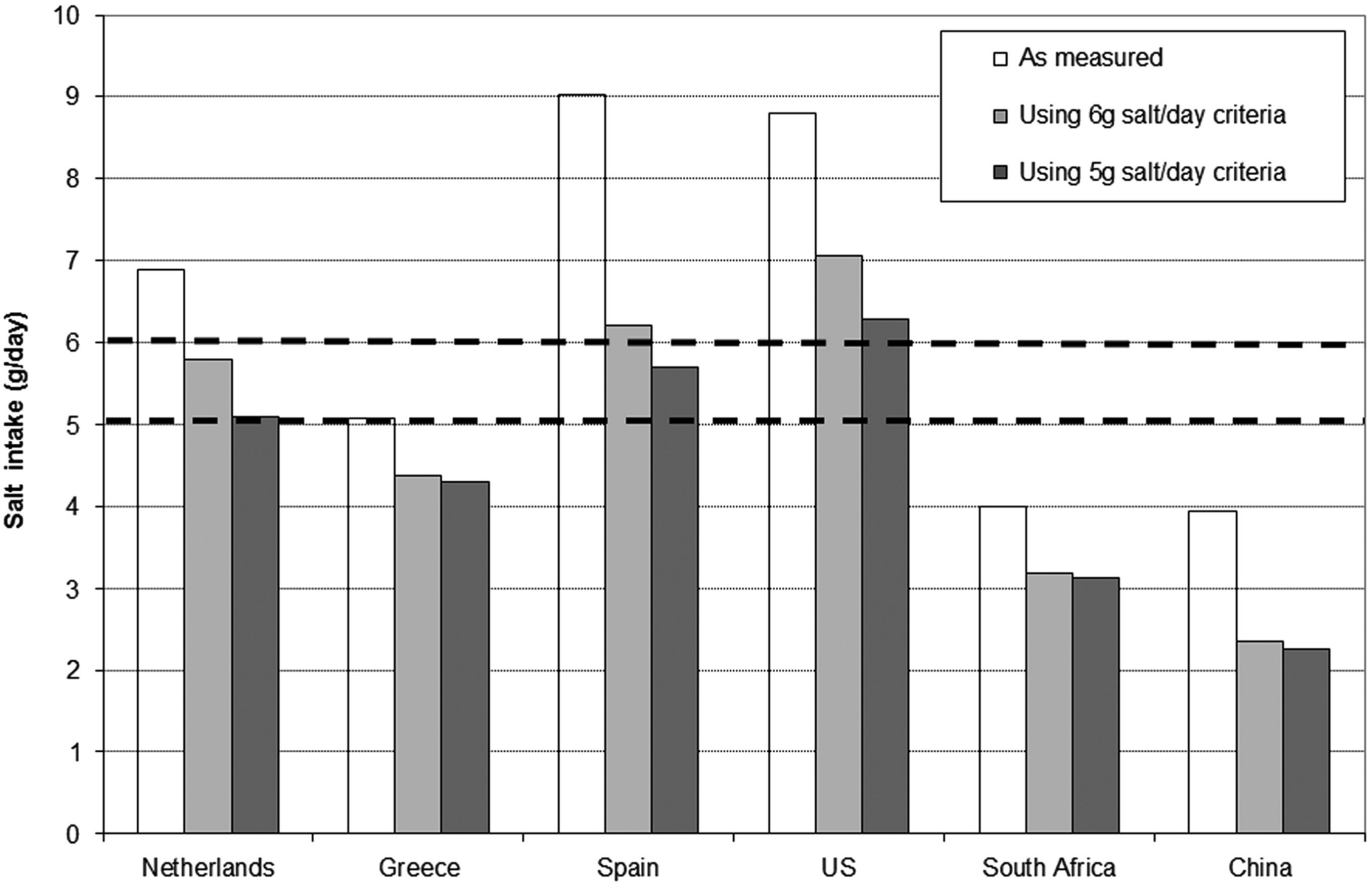
Estimated impact of reformulation on salt intake using daily menu modelling. The vertical bars indicate current salt intake from foods* in the typical daily diet, and the modelled intake after replacing actual sodium levels in food products by 6 and 5 g/day criteria. The horizontal dashed lines indicate total daily salt-intake targets of 6 and 5 g salt/day. *Discretionary salt use was not taken into account.

Iterative modelling by using the typical daily menus showed that if the finally proposed product group-specific sodium criteria would be achieved, it should be possible to move salt intakes towards the interim target of 6 g and the long-term target of 5 g per day. However, the expected impact of salt reduction in food products is different between countries, as shown in Figure 1. In countries such as Spain and the US, reformulation towards the product group-specific sodium criteria may not be sufficient, and increasing consumer awareness on the need to reduce intake of food products that contribute most to their salt intake is required. In China and South Africa, the contribution of food products to overall salt intake is smaller than the other countries (4.0 and 3.9 g/day salt, respectively), and excessive intake is still mainly driven by the discretionary salt that consumers add during food preparation or at the table (3.6 and 10.9 g/day salt, respectively).^19, 20^ Therefore, in these countries, it will also be very important to convince the population to use less discretionary salt.

### Impact evaluation of final criteria on population salt intake (step 2)

**Figure 2:**
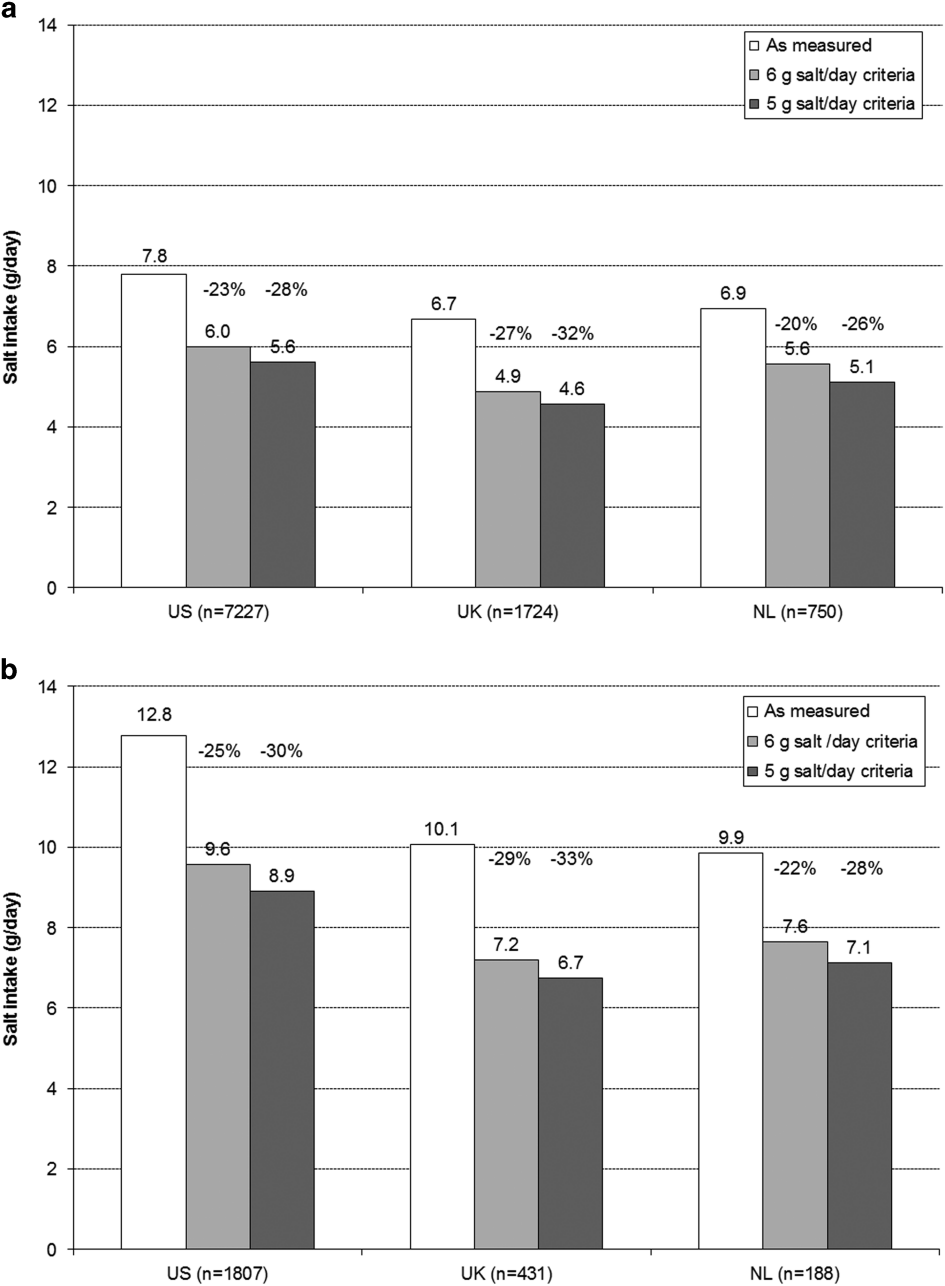
Estimated impact of reformation on salt intake by using the dietary survey data. (**a**) In total population. (**b**) In high-salt users. Median daily salt intake in the total US, UK and NL population at baseline ‘as measured' and the estimated intake after simulated product reformulations according to the 6 and 5 g/day criteria. The percentages indicated reflect the potential relative reduction in total daily salt intake owing to reformulation compared with ‘as measured' intake.

Median daily salt-intake values from food products as measured in the three surveys were 7.8, 6.7 and 6.9 g for populations in the US, UK and NL, respectively (Figure 2). In all three populations, less than 25% of the population had median salt-intake levels compliant to the recommended intake of 5 g salt/day (Table 3). In high-salt users, median daily salt intake was 12.8, 10.1 and 9.9 g per day for the US, UK and NL, respectively (Table 3).

In Figure 2a, the potential impact on salt intake of product reformulation to meet the sodium criteria is shown. Modelling with the 6 g/day criteria yielded a daily salt-intake reduction of 1.4–1.8 g, equalling a 20–27% reduction compared with baseline. The 5 g/day product criteria would result in reductions in salt intake of 1.8–2.2 g per day (26–32%). As expected, results for high-salt users show greater absolute sodium intake reductions (Figure 2b). In this subgroup, salt intake would be reduced with 2.2–3.2 g per day (equalling a 22–29% reduction) using the 6 g/day product criteria and with 2.7–3.9 g (equalling a 28–33% reduction) using the 5 g/day product criteria.

Applying the 6 g/day product criteria would increase compliance with the 6 g/day recommendation in all three populations by at least 25%. Applying the 5 g/day product criteria, the number of people complying with the 5 g/day intake recommendation would more than double in all three countries analysed (Table 3).

## Discussion

In the absence of globally applicable and accepted sodium reduction criteria, the criteria described in this article could serve as a direction for salt reduction in food products. Salt reduction must be seen as a journey—significant changes are required and it will take time to successfully implement. Although it will be very challenging, for technological and/or taste reasons, to get all products fully compliant, the product group-specific sodium criteria could certainly help guide reformulation and reduce population salt intake.

The daily menu modelling was a first step to get insight into the potential impact of the sodium reduction criteria on total salt intake in various countries. However, there were differences in the measurement of salt intake between the countries. For most countries, salt intake was based on the dietary records,^17, 19, 20, 23^ which generally do not properly include the use of discretionary salt. However, for Spain, salt intake was based solely on 24 -h urine excretion,^16^ which does include discretionary salt use. For US, salt used during cooking was included in the recipes of prepared foods.^18^ Therefore, results between countries may not be completely comparable. Another limitation is that the menus reflected a manual translation of nutrient intakes in a country in three typical menus. Therefore, additional robust modelling was performed by using the dietary food consumption survey data from the United States, United Kingdom and the Netherlands. Baseline salt-intake levels significantly exceeded the international guidelines for salt intake for all three populations, indicating the relevance of salt reduction in these countries. In all three populations, modelling with 6 g/day criteria resulted in an average reduction in population salt intake of ~25% modelling with 5 g/day criteria led to an average reduction in population intake of 30%. As a result of cross-industry product reformulations to these criteria, the number of people complying with the recommendation of 5 g salt/day could be more than doubled.

We realise that the modelling is subject to a few limitations that need to be addressed. First, we could not use the most recent surveys for the three countries analysed, as the data were not yet available at the time that the analyses were performed. To get the most up-to-date insight on the potential impact on actual intakes, it would be preferable to repeat the modelling with more recent data that have become available in the meantime. Nevertheless, also publications regarding the more recent survey data indicate that there is still a need for further salt reduction in all three countries examined.^28, 29, 30^

Second, owing to the limited availability of food consumption survey data, the modelling was applied to only three Western countries. To get more insight into the potential impact beyond these countries, the modelling could be expanded to other countries. However, in non-Western countries such as China and South Africa, the contribution of food products to overall salt intake is smaller than in Western countries (27% and 53%, respectively), and excessive intake is mainly driven by a high discretionary salt use.^19, 20^ Therefore, the impact of food reformulation on total salt intake would be much smaller than the 25–30% we estimated for the three Western countries.

Third, a limitation of dietary surveys in general is that these do not properly measure discretionary salt intake. Reductions in total salt intake thus mainly reflect the impact of reformulation of food products. If discretionary salt could have been taken into account, the intake goals of 6 and 5 g would not have been met entirely. This also means that the absolute compliance to the recommendations after simulated reformulation shown in this study is overestimated. Nevertheless, the relative increase in compliance gives a valid reflection of the potential impact of the food reformulation. An additional reduction of discretionary salt use may be achieved by increasing consumer awareness around salt, which should eventually lead to a change in the behaviour. A recent study showed that the number of people in England adding salt to food at the table fell by more than a quarter in the 5 years following a national campaign.^31^

Finally, we acknowledge that the modelling represents a theoretical approach based on dietary survey data with its limitations, and it reflects the results of a best case scenario, assuming that the entire food industry would apply the criteria and all food products would be reformulated to these targets. Although this may not be completely realistic in practice, we feel that the modelling results give a reasonable indication of the impact on population salt intake that could be achieved by food reformulation towards the described product group-specific criteria.

Within Unilever, the criteria are genuinely used to guide salt reduction within the product portfolio. Reductions of up to 25% were made across the entire product portfolio, while maintaining great tasting products. By the end of 2012, more than 50% of the foods portfolio met the 5 g salt/day criteria.^32^ However, salt reduction cannot be limited to product reformulation. With proposed criteria we are already challenging the boundaries of what is technologically possible, but also with regard to consumer acceptance.^2^ Some older studies have shown^33, 34^ that participants did not fully compensate via the salt shaker for the reduced salt content of their diet or meal. However, a more recent study showed that when a single product (soup) was 30% reduced in salt, the majority of the participants did add salt back, even to the point of over-compensating.^35^

In achieving a population salt intake of maximally 5 g/day, there is an important role for health authorities and salt-interest groups to help increase the consumer awareness on the need to reduce salt. Together, Unilever and the International Union of Nutrition Societies initiated consumer studies and stakeholder workshops in Germany/Austria, Hungary, South Africa, China, India and Brazil. The studies revealed that, although salt reduction is seen as relevant to health, the majority of the respondents believed their salt intake was satisfactory, and they were not planning or considering reducing their salt intake. The workshops served as first step in engaging the relevant stakeholders in jointly increasing the consumer awareness on the need to reduce salt intakes, and developing exciting behaviour change approaches in the area of salt reduction.^36^ We are convinced that salt-intake reduction is a shared responsibility of health authorities, salt-interest groups, consumers and also food industry. It should be induced both via product reformulations and behaviour change. We do not agree with the review by Moodie *et al.*^37^ and other articles that are sceptical about the role of food industry in formation of policy regarding non-communicable diseases. The significant decline in salt intake observed in the UK since 2003^30^ is reflecting the successful collaboration between the government and the food industry.^11^ Only when all parties and their actions are joined, successful salt reduction can be stipulated.

In conclusion, the product group-specific sodium criteria described in this paper can serve as guidance for salt reduction in food products. Dietary impact modelling shows that product reformulation can contribute substantially to salt-intake reduction, provided that a cohesive industry-wide approach is taken. Even a small reduction in population salt intake has a considerable health impact. According to meta-analyses, a conservative estimate indicates that a reduction of 1 g/day would result in reduction in blood pressure of 0.8/0.5 mmHg, which would reduce stroke risk by 5% and ischaemic heart disease risk by 3%.^38^ Altogether, many small actions can make a big difference.

## Figures and Tables

**Table 1:** Product group-specific sodium criteria

	*Sodium criteria aiming at*	
	*6 g salt/day (2400 mg sodium/day)*	*5 g salt/day (2000 mg sodium/day)*
Generic criteria	1.6 mg/kcal 100 mg/100 g	1.3 mg/kcal 100 mg/100 g
Bread and breakfast cereals	375 mg/100 g	375 mg/100 g
Sandwiches/rolls	1.6 mg/kcal	1.4 mg/kcal
(Un)processed meat, meat products, meat substitutes	675 mg/100 g	675 mg/100 g
(Un)processed fish, fish products	340 mg/100 g	340 mg/100 g
Cheese (products)	900 mg/100 g	675 mg/100 g
Main dish	1.6 mg/kcal	1.6 mg/kcal
Side dishes	250 mg/100 g	250 mg/100 g
Meal sauces	540 mg/100 g	340 mg/100 g
Dressings, emulsified (e.g., mayonnaise)	1080 mg/100 g	750 mg/100 g
Dressings, water-based (e.g., ketchup)	1080 mg/100 g	750 mg/100 g
Seasonings (e.g., herbs and spices, marinades)	360 mg/100 g	265 mg/100 g
Soups and bouillons	360 mg/100 g	265 mg/100 g
Snacks (sweet and savoury, incl. ice-cream)	1.6 mg/kcal or 100 mg/100 g	300 mg/100 g
Spreads and cooking products (margarine, butter and oil/fat products for roasting and frying)	1.6 mg/kcal or 720 mg/100 g	1.3 mg/kcal or 470 mg/100 g

**Table 2:** Overview of food consumption survey and composition tables used in assessments

*Food consumption survey*	*Composition table*	*Sample size (*n*)*	*Population age*	*Data collection method*
NHANES 2007-2008	FNDDS 4.1 2008	7227	Total population 2–80 yrs	2-Day 24- h recall + specific questionnaire (to enable recipe calculation)
NDNS 2000-2001	McCance and Widdowson's 5th edition	1724	Adult population 19–64 yrs	7-Day food diary + blood and 24-h urine samples
DNFCS 2003	NEVO 2001 in combination with 2006	750	Young adults 18–30 yrs	2-Day 24- h recall by dieticians+general questionnaire

**Table 3:** Compliance to 6 and 5 g salt/day intake goals at baseline and after simulated product reformulations

*Scenario description*	*Overall population compliance with intake goal (%)*	
	*6 g/day*	*5 g/day*
*USA*		
‘As measured' salt intake	27	15
Applying 6 g salt/day criteria	50	33
Applying 5 g salt/day criteria	57	39
		
*UK*		
‘As measured' salt intake	39	20
Applying 6 g salt/day criteria	74	55
Applying 5 g salt/day criteria	80	62
		
*NL*		
‘As measured' salt intake	35	22
Applying 6 g salt/day criteria	60	40
Applying 5 g salt/day criteria	69	49

Abbreviations: UK, United Kingdom; US, United States; NL, Netherlands.

## Notes

All authors are employed (MD-K, WPMMG and KHvhH) or were employed at time of the study (GWM) by Unilever Research and Development Vlaardingen, the Netherlands.
