# Supplementary material for: Reducing salt in food; setting product-specific criteria aiming at a salt intake of 5 g per day
Source: Eur J Clin Nutr. 2015 Feb 18;69(7):799–804. doi: 10.1038/ejcn.2015.5 (PMC4493648; doi:10.1038/ejcn.2015.5)
Supplement: Supplementary Information [file ejcn20155x1.doc]

**Supplementary Information**

**Methods Daily Menu modelling**

Product group specific sodium criteria were tested using typical daily menus. For several countries, average salt intake levels from national surveys were converted into three typical Daily Menus per country. To provide a global representation, daily menus were created for the Netherlands, Spain, Greece, USA, South Africa and China. Nutrition surveys produce nutrient intake data that are representative for the study population. Usually, information on foods most commonly consumed is included as well. All this information was used to compose three Typical Daily Menus, representative for the dietary intake of a healthy adult. The daily menus were composed manually by a nutritionist or dietician who knew the dietary habits of the country’s population, using an iterative process in order to get the nutrient intakes from the menu’s as close as possible to the survey results**.** The maximum deviation allowed was 20%.

The table below gives the details of dietary intake data used to compose the Daily Menus. Salt intake levels used for the modeling did not include intake from discretionary salt, except for Spain where salt intake could be based solely on 24h urine excretion, and the US where salt used during cooking was included in the recipes of prepared foods. For China and South Africa, we have only used information on the urban populations as these menus contain relatively less homemade dishes and more processed foods compared to menus in rural populations. However, it is clear that in both these countries salt intake is still driven mainly by discretionary salt rather than intake with foods. Discretionary salt intake in China was reported to be 10.9 g/day for the urban population (18). For South Africa reported intake of discretionary salt was 3.6 g/day (17). For the other surveys we do not have a good insight into discretionary salt intake, but it has been estimated that in Western countries over 75% of salt intake comes from processed foods (3;4). For more details regarding the development of the Daily Menus see Roodenburg et al. (16).

Table Overview of nutrient intake data sources for the various countries

| **Country** | **Survey** | **Year** | **Study population** | **Dietary assessment method** |
| --- | --- | --- | --- | --- |
| **Netherlands** | National Survey | 1998 | All age groups (n=5958) | 2 day dietary record |
| **Greece** | Survey university of Crete | 1989-2001 | Medical students  aged 20-24  (n=951) | 1 day 24h recall |
| **Spain** | Intersalt study | 1986 | Adults  aged 20-59  (n=400) | 24h urinary sodium excretion |
| **US** | National Health and Nutrition Examination Survey | 1999-2000 | All ages  (n=8604) | 1 day 24h recall |
| **China** | National Survey | 2002 | Urban population  all age groups  (n=21103) | 3 day 24h recall;  food-weighted record on household level |
| **South Africa** | Study on diet and blood pressure | 2002 | Black urban adults  aged 20-65  (n=110) | 24h urinary sodium excretion |

Using these menus, the impact of product group specific sodium criteria on total daily salt intake was estimated crudely by adjusting sodium levels of all non-complying products in these menus to the levels of the test criteria. Finally, the average total daily salt intake after adjusting the sodium product values was compared with salt recommendations to evaluate the effectiveness of the sodium test criteria. This iterative process finally led to the 6 g salt/day and 5 g salt/day product group criteria. The Daily Menu Modelling is visualized in Figure 1.


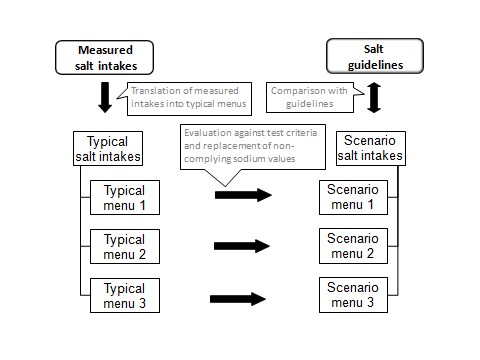


**Figure Schematic overview of the Daily Menu method**

**Rationales behind the 6 g salt/day and 5 g salt/day product group criteria**

*6 g salt/day – sodium criteria*

Daily menu modelling revealed that with applying the sodium criteria of the initial Nutrition Enhancement Programme it would be possible to reduce population salt intake towards the interim target of 6 g salt/day. Therefore, these criteria were taken as 6 g salt/day criteria.

In principle, generic criteria were applied, which is based on direct transformation of the dietary guidelines, i.e. 2400 mg sodium per 2000 kcal = 1.2 mg/kcal. However, many food products contain much less than 1.2 mg/kcal (e.g. fresh/frozen fruit & vegetables, milk products, beverages), leaving room for more sodium in other food products. Therefore, the criterion was set at 1.2 mg/kcal +30% = 1.6 mg/kcal. For low-calorie products, a criterion in mg/kcal would be unrealistic. Therefore, a so-called insignificance level was set in addition. This insignificance level was set at ≤100 mg sodium /100 g food product. Generic criteria were applied to: sandwiches/rolls, main dish, snacks, spreads and cooking products, and all other products for which no group specific sodium criteria were set.

Product group specific sodium criteria were set for products where generic criteria were inappropriate. Product group specific criteria were set pragmatically, but taking the following aspects into consideration:

- criteria in existing local nutrient profiling systems;
- a thorough understanding of product composition;
- the role of the food product in the diet;
- iterative modeling using the Daily Menus

The existing product specific sodium criteria of the Nutrition Enhancement Programme were applied for: cheese (products), meal sauces, dressings (water and emulsion based), soups and bouillons, spreads and cooking products. For more details see Nijman et al. (6).

Importantly, the Nutrition Enhancement Programme did not include specific sodium criteria for bread and breakfast cereals, meat products, fish products, snacks, side dishes (carbohydrate based products with sauce or seasoning) and seasonings (e.g. spice mixes, marinades, soy sauce). Following the total diet approach, additional product specific sodium criteria were defined for these product groups (see below).

*5 g salt/day – sodium criteria*

The energy-based generic criterion was calculated the same way as for the 6 g salt/day criterion but taking the WHO recommendation of 5 g salt (2000 mg sodium) as basis: 2000 mg sodium / 2000 kcal = 1.0 mg/kcal + 30% = 1.3 mg/kcal. The insignificance level was kept at the same level of ≤100 mg sodium /100 g food product. For defining the 5 g salt /day product group specific sodium criteria, the international Choices criteria have served as a basis (11).

The principle used for the Choices criteria was that consumers should have access to more healthy choices within basic product groups—as these provide essential nutrients—than within the non-basis product groups. For basic product groups, the aim was to have at least 20% of products comply with the criteria within a given product group, and approximately 10% for the non-basic product groups. In addition, factors such as public health nutrition, food market reality (actual foods on the market) and the typical examples of indicator foods were used during the scientific debates to further define the criteria (11).

The daily menu modelling revealed that the Choices criteria would not be strict enough to enable a total daily intake target of 5 g salt/day. Therefore, the criteria were adjusted iteratively until the average modelled daily intake over the six countries used in the daily menu modelling was reaching the target intake of 5 g salt/per day.

More detailed information for product specific sodium criteria not included in the Nutrition Enhancement Programme and/or Choices Programme or where adaptation of the general approach was necessary is provided below.

*Bread and breakfast cereals, meat products and fish products*

The Nutrition Enhancement Programme did not include product group specific sodium criteria for these product groups, but these groups generally contribute substantially to daily salt intake (>20%). Daily menu modelling revealed it was necessary to set quite strict criteria in order to enable a total daily salt intake of 6 g salt per day. Therefore for these product groups, the 5g salt/day criteria were also applied as 6 g salt/day criteria.

*Side dishes*

A prepared side dish generally contains about 65% of a carbohydrate component and 35% of meal sauce. Both the Nutrition Enhancement Programme and Choices Programme do not include a product group specific sodium criterion for side dishes, but the generic criteria do not seem feasible. Therefore, the generic sodium criterion (as applied for grains and vegetables) (100 mg/100g) and the criterion for meal sauces (540 mg/100g) were combined in the ratio 65%:35%; i.e. 65% of the criteria defined for carbohydrates and 35% for meal sauces are combined: (0.65 x 100 mg sodium/100g) + (0.35 x 540 mg sodium/100g). This provided a sodium criterion for side dishes of 250 mg sodium/100g (as prepared). Viewing current sodium values on the market this is very strict already. Therefore, the criterion is used both in 6 g salt/day and 5 g salt/day criteria.

*Seasonings*

Seasonings include herbs, spices and spice mixes, marinades and tenderisers. Both the Nutrition Enhancement Programme and the Choices Programme do not include a product group specific sodium criterion for side dishes, but also for these products the generic criteria do not seem feasible. The sodium content per 100 gram of seasoning ‘as sold’ is very different between the various types of seasonings. In the prepared product the difference in sodium content is less significant. This also gives a more realistic view on how much sodium the consumer takes in when consuming a seasoned dish. Therefore, the criterion was set per 100g of prepared product.

The sodium content of seasonings as prepared per 100g product was calculated using current ‘as sold’ sodium levels in seasonings and available habitual dosing/current dosing instructions. Furthermore, it was decided that the sodium contribution of 2-3 different seasoned dishes to a meal should not be more than the sodium criterion for a main dish. Taking the current sodium levels and contribution to a meal into account the following criteria were defined (in line with soups and bouillons):

- - 6 g salt/day criterion: 360 mg per 100 g of prepared product
  - 5 g salt/day criterion: 265 mg per 100g of prepared product

*Spreads and cooking products*

In the Choices Programme spreads and cooking products have to comply with the generic energy-based sodium criterion of 1.3 mg/kcal. However, in order to set a realistic sodium criterion for reduced fat variants, a 5 g salt/day criterion in mg/100g was defined in addition to the energy-based criterion of 1.3 mg/kcal. The criterion was calculated as follows.

- The energy-based criterion in en% is converted to a criterion in mg/100g
- 40% fat spreads are taken as the standard: 40 * 9 = 360 kcal/100g
- The energy-based 5 g salt/day criterion is 1.3 mg/kcal: 360 *1.3 = 470 mg/100g

*Dressings and table sauces:*

In the Choices Programme the sodium criterion for water- and emulsion based sauces (e.g. ketchup and mayonnaise) was set at 750 mg/100g (11). Reducing the criterion even further would cause a serious preservation issue in low calorie variants. Therefore, the 5 g salt/day criterion was kept at 750 mg/100g.

*Note: Snacks*

For snacks there may seem to be a discrepancy between the product group specific 6 g salt/day and 5 g salt/day criteria, but this due to the fact that the 6 g salt/day criteria are based on the Unilever Nutrition Enhancement Programme, which did not include product group specific criteria for snacks, whereas the 5 g salt/day criteria are based on the criteria of the Choices Programme, which does include product group specific criteria for snacks.
